# Characterizing trends in HIV infection among men who have sex with men in Australia by birth cohorts: results from a modified back-projection method

**DOI:** 10.1186/1758-2652-12-19

**Published:** 2009-09-18

**Authors:** Handan Wand, David Wilson, Ping Yan, Andrea Gonnermann, Ann McDonald, John Kaldor, Matthew Law

**Affiliations:** 1 National Centre in HIV Epidemiology and Clinical Research, Sydney, Australia; 2 Center for Infectious Disease Prevention and Control Population and Public Health Branch, Canada; 3 Human and Health Sciences, University of Bremen, Germany

## Abstract

**Background:**

We set out to estimate historical trends in HIV incidence in Australian men who have sex with men with respect to age at infection and birth cohort.

**Methods:**

A modified back-projection technique is applied to data from the HIV/AIDS Surveillance System in Australia, including "*newly diagnosed HIV infections*", "*newly acquired HIV infections*" and "*AIDS diagnoses*", to estimate trends in HIV incidence over both calendar time and age at infection.

**Results:**

Our results demonstrate that since 2000, there has been an increase in new HIV infections in Australian men who have sex with men across all age groups. The estimated mean age at infection increased from ~35 years in 2000 to ~37 years in 2007. When the epidemic peaked in the mid 1980s, the majority of the infections (56%) occurred among men aged 30 years and younger; 30% occurred in ages 31 to 40 years; and only ~14% of them were attributed to the group who were older than 40 years of age. In 2007, the proportion of infections occurring in persons 40 years or older doubled to 31% compared to the mid 1980s, while the proportion of infections attributed to the group younger than 30 years of age decreased to 36%.

**Conclusion:**

The distribution of HIV incidence for birth cohorts by infection year suggests that the HIV epidemic continues to affect older homosexual men as much as, if not more than, younger men. The results are useful for evaluating the impact of the epidemic across successive birth cohorts and study trends among the age groups most at risk.

## Background

After a steady decline since the mid 1980s, there is now growing evidence that HIV infection has been increasing in parts of the developed world [1]. Despite apparent successes in the past, the most current data [2] indicate an ~40% increase in new HIV diagnoses in 2007 from the year 2000 among Australian men who have sex with men (MSM).

Increases over time in the epidemic could potentially reflect the risk profile of new generations of MSM as they become sexually active. Therefore, describing the trends in HIV incidence by year of infection for successive birth cohorts can potentially provide a comprehensive picture of the epidemic. Comparing incidence estimates obtained from these sub-populations (e.g., "youngest" versus "oldest") can be fundamental in providing indications of the epidemic trends in terms of demography for informing public health responses.

Australia established an HIV surveillance system in the early 1980s, whereby all new HIV diagnoses are reported. Since 1991, further surveillance has been supplemented by national notification of HIV diagnoses with evidence of newly acquired HIV infection, defined as new HIV diagnoses with either a previous negative HIV test within 12 months, or with evidence of a recent seroconversion illness. Although these data are indicative of trends in the HIV epidemic, they cannot be used directly to estimate HIV incidence (number of new infections per year). Accurate estimates of HIV incidence by population subgroup are required to determine trends in the epidemic and to evaluate the groups most at risk for acquiring HIV.

Methods based on back projection [3] have historically been used to estimate HIV incidence from AIDS surveillance data based on an assumed probability distribution for the incubation period, that is, the time from HIV infection to AIDS. The availability of effective antiretroviral therapies since 1997 has altered the distribution of the incubation period in ways that are difficult to quantify. The current study used a modified back-projection methodology to estimate the annual number of HIV infections by using the HIV/AIDS surveillance data to reconstruct the HIV epidemic among successive birth cohorts since the onset of the epidemic.

This method allows us to obtain estimates of the past and current incidence trends in a population over each calendar year and with respect to age at infection. Similar approaches have been used to estimate age-specific historical trends in the past [4-7], but the methodology used in this study extends previous methods and makes maximal use of available HIV/AIDS data sources in Australia's surveillance databases, including "*newly diagnosed HIV infections*", "*newly acquired HIV infections*" and "*AIDS diagnoses*", to estimate trends in HIV incidence.

Since there is no established statistical model to link HIV incidence to HIV diagnosis with respect to HIV testing patterns, the current methodology assumed that if an individual was infected before, or in, a certain year, it was more likely that this individual sought an HIV diagnostic test at the onset of clinical symptoms. However, as HIV testing became more available and promoted, individuals infected in later years tended to be more likely to seek testing independent of the onset of clinical symptoms. Unlike the similar models used in the literature [4] where both "time at HIV diagnoses" and "time at AIDS diagnoses" data have almost equal roles, in our approach, "time of HIV infection" is the predominant variable, whereas "time at AIDS diagnosis" data is less influential.

This methodology is applied to Australia's HIV/AIDS National Surveillance data to estimate the number of HIV infections among MSM and to evaluate the impact of the year of birth and age at infection. It is also used to assess whether incremental increases in age at diagnoses [2] in recent years truly reflect increases in age at infection.

## Methods

In Australia, HIV transmission is monitored through the notification of cases of newly diagnosed HIV infection, including cases with evidence of newly acquired HIV infection (which is defined as HIV infection with evidence of a prior negative test, a diagnosis of primary HIV infection, or an indeterminate western blot within 12 months of HIV diagnosis). There are potentially three data sources of HIV surveillance data available in each calendar year: new HIV-positive diagnoses by year of diagnosis; newly acquired HIV infection (recent infections among new HIV diagnoses); and new AIDS cases (based on physicians' reporting on diagnoses of clinical events subject to AIDS Case Definition) [2].

### Modified back-projection method by birth cohorts

The back-projection method was originally proposed by Brookmeyer and Gail and used in western countries in the late 1980s and early 1990s to estimate trends in HIV infections based on reported AIDS diagnoses [3]. Later studies modified the back-projection methodology to account for the increased incubation period due to treatment [8,9].

The method used here differs from similar approaches in the literature in that it does not require data linkage between the HIV and AIDS diagnostic registries. It is based on a parametric formulation of the duration of time between the time of acquisition of HIV infection and the time of earliest diagnosis of HIV infection from enhanced HIV surveillance systems or from laboratory confirmed testing. Many factors may influence this distribution (e.g., awareness of recent exposure due to onset of symptoms related to the HIV progression process, random detection due to accidental circumferences, or frequent testing), but in this study we consider two testing "forces" as described below.

### Sub-model 1: HIV testing during asymptomatic infection

A proportion of people infected with HIV will be diagnosed with HIV prior to clinical symptoms or AIDS. A heterogeneous mixed exponential model was used to model the rate at which people in this group are diagnosed with HIV, assuming a constant testing rate *λ*. This leads to a Pareto distribution with decreasing hazard function for the duration *X *between HIV infection and HIV diagnosis, which essentially steps down over time. The survivor and hazard functions are

We define

for the probability of testing *x *= 0, 1, 2,... years after infection.

### Sub-model 2: HIV testing driven by clinical symptoms at late stage of HIV progression

A proportion of HIV diagnoses are assumed to be made at a late stage of HIV infection or at AIDS diagnosis. We assumed that the progression from HIV infection to the earliest HIV diagnosis follows a distribution similar to the progression to CD4 counts of <200 cells/mm^3 ^without any treatment. A Weibull distribution was adopted, with median time to HIV diagnosis of 6.5 years and shape parameter 2.08 [10] with the survivor and hazard functions:

We define

The Weibull distribution has the property that the hazard increases with time from infection, which intuitively would mirror the risk of progression to HIV-related symptoms in untreated HIV infection.

### Overall rate of progression to HIV diagnosis

The overall rate of progression to HIV diagnosis *f*(*x*|*t*; *φ*) was formulated by combining the two sub-models (i.e., *f*_*a*_(*x*) and *f*_*b*_(*x*)) described above using a mixture distribution model, i.e.,

where S(*x*|*t*; *φ*) is the survival function:

where , *t *≥ *t*_0 _is a mixing function with *φ *= (*π*, *δ*, *γ*); *π *represents the proportion of infected individuals who were not tested because of clinical symptoms; *δ *determines the overall shape of the curves; and γ denotes the rate of increase in infection at time *t*.

When HIV testing just became available at *t *= *t*_0_, , as *m*_*t*_(*φ*) increases with time *t *at rate *γ *to a saturation level *π *= lim_*t *→ ∞ _*m*_*t*_(*φ*) We assume that there will be a proportion 1 - *π *of infected individuals who are driven by clinical symptoms to be tested (as specified by Sub-model 2).

**Figure 1:**
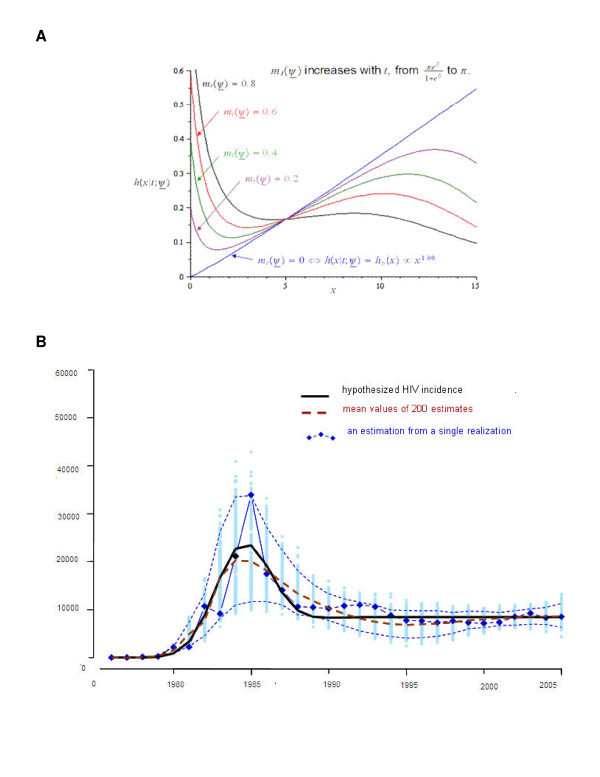
**Sensitivity analysis**. **(A)**. Shapes of hazard function of the mixture model, *h*(*x*|*t*; *φ*) for various values of *m*_*t*_(*φ*). **(B)**. Results from simulation study: the mean values of the HIV incidence estimates from 200 realizations are shown with the broken red line; the hypothetical HIV incidence is shown with the black solid line.

The mixture distribution model *f*(*x*|*t*; *φ*) results in an overall "bath-tub" shaped hazard (Figure 1a). This model was allowed to vary over time, so that the proportion of diagnoses due to clinical symptoms was assumed to decrease over time. The combination of the two distributions was left truncated at 1985, prior to the availability of HIV testing in Australia when HIV diagnosis was only made on the basis of AIDS symptoms.

The HIV incidence curve was then reconstructed by combining two back-projection estimated HIV incidence curves from AIDS diagnostic data (up to 1994, prior to which effective antiretroviral treatment was not available) and HIV diagnostic data using the combined progression rate distribution.

This model uses three key parameters: the proportion of infected individuals who are "late testers" (from Sub-model 2); the rate of increase in incidence over time; and a shape parameter. The final estimates for HIV incidence were produced using an optimization method based on Nelder-Mead, quasi-Newton and conjugate-gradient algorithms written in R-language.

### Sensitivity analysis

Sensitivity analyses were performed to assess the robustness of the hypothetical HIV incidence curve for a variety of shape parameters. Figure 1a presents the hazard function h(*x*|*t*; *φ*) as defined by *m*_*t*_(*φ*) at the values of 0.2, 0.4, 0.6 and 0.8 for the shape parameter.

The properties of the parameters used in this approach were also studied with a series of simulation studies. We predicted the mean numbers of HIV diagnoses over time using a variety of parameter estimates. We used the log-logistic distribution with mean = 10 years and a shape parameter 3.08 for the HIV to AIDS incubation period to predict the mean numbers of new AIDS diagnoses up to the time of the availability of effective antiretroviral therapies in 1997. We set the testing behaviour parameter to 0.5 per year before we predicted the proportions of recent infections.

For each year, we generated 200 random numbers for each time point from a mixed-Poisson distribution around the predicted mean values with an over-dispersion factor to account for the extra-Poisson variation. The resulting 200^3 ^sets of simulated data from all parameter combinations contained: (i) the primary data for HIV diagnoses; (ii) the AIDS diagnostics data to adjust for the ramp-up period; and (iii) the auxiliary data for proportions of recent infections among newly diagnosed cases. Results are presented in Figure 1b.

### Application to the Australian birth cohorts

To explore the possible effects of birth cohorts on annual HIV incidence over time, birth years were grouped in 10 five-year birth cohorts; in addition, men born after 1980 or men born before 1940 were grouped together. Changes in the age distribution of HIV infections over time were examined by considering "age at diagnosis" and "age at infection". Age at diagnosis was obtained directly from available data on the date of person's new HIV diagnosis and year of birth.

**Figure 2:**
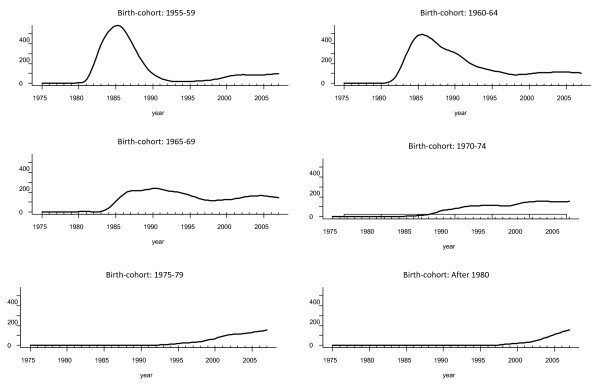
**Trends in estimated HIV infections (from back-projection method) by birth cohorts**.

Our modified back-projection method was then applied to each birth cohort separately to produce a matrix for HIV infections with rows as each calendar year at infection and columns with birth cohorts. Rotating this matrix by 45 degrees produced a new matrix, which has the number of infections for each calendar year versus age at infection. One can roughly translate the age group and calendar time of HIV infection into the time of birth for each individual. For example, people who were born from 1960 to 1964 and infected with HIV in 1984 correspond to the age interval for "*20-24 years*". Finally, "mean age at infection" was estimated from the mid-point of each birth cohort and plotted as a smooth function against "year at infection" to investigate possible non-linear associations (Figure 2).

## Results

Following a long-term decline, the annual number of new HIV diagnoses, according to data from Australia's HIV/AIDS surveillance system, has recently been increasing: from 557 cases in 2000 to 774 in 2007. Among newly diagnosed HIV cases, an increasing number were in people who had acquired HIV infection within the previous year. A total of ~21,000 cases of HIV infection were diagnosed by December 2007 among homosexually active men in Australia, which accounted for more than 75% of all reported cases. Sensitivity analyses revealed that the magnitudes and shape of the estimated HIV curves were slightly sensitive to assumptions about the parameters in the models (Figures 1a and 1b). However, the primary qualitative conclusion of the analysis was very robust.

### Patterns of "age at diagnosis" and "age at infection"

Consistent with overall patterns, it is estimated that all age groups experienced a sharp decline in HIV incidence from 1985 until the mid 1990s, and then HIV incidence levelled off between 1995 and 2000 (Figure 2). But since then, there has been a steady increase in incidence across all age groups, with the older age groups experiencing the greatest magnitude and earliest nadir in incidence.

Although age at HIV diagnosis does not tell us when persons were first infected, because their HIV diagnosis may take place several months or years after infection, it is frequently used to describe the epidemic among different populations. Here, we also estimated the age at infection for each calendar year. Table 1 presents the mean age at diagnosis (observed) and the mean age at infection (estimated by back-projection method) by calendar year.

**Table 1:** Summary of age at infection and age at diagnosis by calendar year

	Mean age	
Calendar year	At infection (back calculation) age ± se^1^	At diagnosis (observed) age ± se^1^
**1985**	31.21 ± 0.17	32.31 ± 0.65
**1986**	29.80 ± 0.18	32.44 ± 0.68
**1987**	29.64 ± 0.19	33.86 ± 0.20
**1988**	29.29 ± 0.22	34.61 ± 0.24
**1989**	28.34 ± 0.24	34.43 ± 0.25
**1990**	27.42 ± 0.26	34.03 ± 0.28
**1991**	27.11 ± 0.28	34.41 ± 0.28
**1992**	27.23 ± 0.29	35.58 ± 0.32
**1993**	27.67 ± 0.30	34.65 ± 0.34
**1994**	28.59 ± 0.31	35.49 ± 0.36
**1995**	29.92 ± 0.31	36.00 ± 0.37
**1996**	31.51 ± 0.31	36.19 ± 0.38
**1997**	33.10 ± 0.32	37.00 ± 0.41
**1998**	34.12 ± 0.31	37.60 ± 0.46
**1999**	34.68 ± 0.30	36.90 ± 0.45
**2000**	35.10 ± 0.29	37.37 ± 0.43
**2001**	35.72 ± 0.27	36.77 ± 0.42
**2002**	36.57 ± 0.26	37.61 ± 0.41
**2003**	37.19 ± 0.25	38.54 ± 0.42
**2004**	37.39 ± 0.25	38.15 ± 0.40
**2005**	37.50 ± 0.23	38.52 ± 0.40
**2006**	37.72 ± 0.19	38.77 ± 0.39
**2007**	37.81 ± 0.27	38.53 ± 0.38

^1 ^Standard error

Overall, it was found that the average age at infection has tended to increase over time since the early 1990s, from ~27 (standard error, se = 0.26) years in 1990, to ~35 (se = 0.29) years in 2000, and ~38 (se = 0.27) years in 2007. Such a trend is suggestive of an increase over time in HIV incidence among older individuals relative to younger individuals. This is reflected by a continual increase in the average age of HIV diagnosis (Table 1).

Although it is expected that the average age at infection would be less than the average age at diagnosis, this difference was pronounced between 1990 and 1999 (approximately six to seven years of difference on average). The difference between the average infection and diagnosis ages is a surrogate indicator for the average time between infection and diagnosis. The difference in average ages of infection and diagnosis has decreased significantly over time, consistent with increases in HIV testing rates [11]. Currently, it is estimated that there is ~1 year difference between average infection and diagnosis ages.

### Patterns of new HIV infections by birth cohorts

HIV incidence was also estimated for each birth cohort and plotted as a smoothed function over calendar years in Figure 2 (birth cohorts before 1954 were not shown). The annual incidence among MSM who were born before 1965 peaked around 1985. Men in this age group were highly sexually active at the beginning of the HIV epidemic. During these peak years, people who were born after 1965 were younger than 19 years old and were at low risk.

However, this younger group subsequently contributed to a rising HIV infection trend after 1985. It is clear that the younger age groups experienced delays in the onset of HIV infections (until they were sufficiently sexually active as expected). But younger age groups also experience longer incidence levels; that is, as age decreases, progressively the duration of the peak is longer and, in fact, the first peak in incidence has now been reached for MSM born after 1970. Figures also indicate that the annual HIV incidence of new infections appears to have reached a plateau between 1995 and 2000 in all birth cohorts, but since 2000, HIV incidence has been steadily increasing in all birth cohorts.

**Figure 3:**
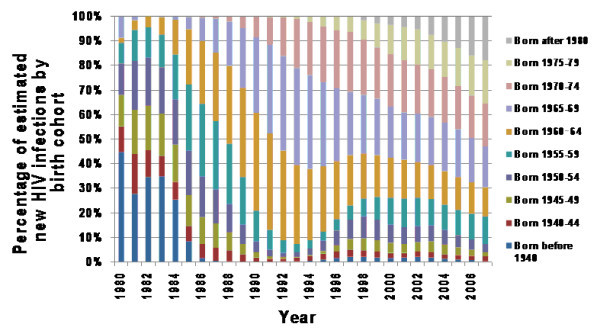
**Distribution of estimated proportion of HIV infections by birth cohort**.

In Figure 3, we present the estimated proportion of all HIV infections by birth cohorts over time. When the epidemic peaked in the mid 1980s, the majority of HIV infections (56%) were attributed to men born after 1955 (who were 30 years or younger); 30% of them occurred in 31 to 40 year olds; and only ~14% of them were attributed to the group who were older than 40 years of age. In contrast, in the year 2000, only ~16% of HIV infections occurred in men younger than 30 years of age, those who were 30 to 40 years old (born between 1960 and 1974) constituted most of the infections (58%), and 26% of all infections were attributed to the group born before 1959 (who were older than 40 years). In 2007, the proportion of infections occurring in persons 40 years or older reached the highest level (31%) since the beginning of the epidemic.

## Discussion

Estimates of past and current incidence of HIV are important for assessing the impact of public health prevention strategies and need to be updated regularly to describe the changing face of the epidemic. Particularly, monitoring new HIV infections among various age groups may provide one of the sources of information on the spread of HIV that can contribute to a better understanding of the HIV epidemic at the national level. Results based on age at infection are useful to assess if the average age of infection is increasing over time, whereas results based on birth cohort are important for understanding demographic trends in HIV infections. Together, they provide considerable insight into the epidemic to inform HIV prevention programmes.

To our knowledge, this is the first study to investigate the impact of age and birth cohorts on an HIV epidemic by using advanced back-projection methodology.

As the epidemic has matured, the primary route of HIV transmission in Australia has remained unprotected homosexual contact. Annual HIV diagnoses and the incidence of HIV infection in Australia during the 1990s fell to levels below the peak of the mid 1980s. Although this decline was seen in all age groups, it was more pronounced in homosexual men older than 30 years, with more modest reductions in age groups younger than 30 years [12].

Using a novel adaptation of a back-projection method, we estimated that the annual incidence of HIV infections among men who have sex with men in Australia has been gradually increasing since the late 1990s, from ~500 new infections per year to 874 new infections in 2007. An increase occurred among all age groups.

These analyses may help in providing greater understanding of the dynamics of the HIV epidemic, based on high-quality surveillance data, and provide reasonably reliable estimates of HIV infection. Our improved methodology has allowed quantitative assessments of the HIV epidemic by birth cohorts, thus providing a sound basis for informing targeted public health policy. This method could also be easily applied to other settings by using the publicly available software written in R-language. Further technical and methodological documents are available upon request ping_yan@phac-aspc.gc.ca.

## Competing interests

The authors declare that they have no competing interests.

## Authors' contributions

HW, PY and DW were responsible for the study concept and design. They also undertook the analysis and interpretation of data. HW and AM extracted the data, and HW drafted the manuscript. All authors undertook critical revision of the manuscript for important intellectual content. All authors read and approved the final manuscript.

## References

[B1] Grulich AE Kaldor JM Grulich AE Kaldor JM Trends in HIV incidence in homosexual men in developed countries Sexual Health 2008 12 113 118 10.1071/SH07075 18588775

[B2] NCHECR HIV/AIDS, viral hepatitis and sexually transmissible infections in Australia Annual Surveillance Report 2008 Sydney, NSW: National Centre in HIV Epidemiology and Clinical Research (NCHECR), The University of New South Wales

[B3] Brookmeyer R Gail MH Minimum size of the acquired immunodeficiency syndrome (AIDS) epidemic in the United States Lancet 1986 12 1320 1322 10.1016/S0140-6736(86)91444-3 2878184

[B4] Becker NG Watson LF Carlin JB A method of non-parametric back-projection and its application to AIDS data Statistics in Medicine 1991 12 1527 1542 10.1002/sim.4780101005 1947509

[B5] Becker NG Watson LF Marschner IC Motika M Newstead SV Carlin JB Assessing the extent of the Australian HIV epidemic from AIDS surveillance data Australian Journal of Public Health 1993 12 226 231 8286495 10.1111/j.1753-6405.1993.tb00140.x

[B6] Rosenberg PS Backcalculation models of age-specific HIV incidence rates Statistics in Medicine 1994 12 1975 1990 10.1002/sim.4780131909 7846404

[B7] Law MG Rosenberg PS McDonald A Kaldor JM Age-specific HIV incidence among homosexually active men in Australia Med J Aust 1996 12 715 718 8668076 10.5694/j.1326-5377.1996.tb122268.x

[B8] Muñoz A Hoover DR Use of cohort studies for evaluating AIDS therapies AIDS Clinical Trials 1995 New York: Wiley 423 446

[B9] Cui J Becker NG Estimating HIV incidence using dates of both HIV and AIDS diagnoses Statistics in Medicine 2000 12 1165 1177 10.1002/(SICI)1097-0258(20000515)19:9<1165::AID-SIM419>3.0.CO;2-7 10797514

[B10] Brookmeyer R Gail MH Generalized back-calculation: extension to account for nonstationary incubation distributions AIDS Epidemiology: A Quantitive Approach 1994 New York: Oxford University Press 219

[B11] NSW, VIC and QLD Gay Periodic Surveys 1999 Sydney, Australia: National Centre in HIV Social Research, University of New South Wales

[B12] Law MG Rosenberg PS McDonald A Kaldor JM Age-specific HIV incidence among homosexually active men in Australia Medical Journal of Australia 1996 12 715 718 8668076 10.5694/j.1326-5377.1996.tb122268.x

